# Ultra-narrowband dielectric metamaterial absorber with ultra-sparse nanowire grids for sensing applications

**DOI:** 10.1038/s41598-020-58456-y

**Published:** 2020-01-30

**Authors:** Yan-Lin Liao, Yan Zhao

**Affiliations:** 1 Key Lab of Opto-electronic Information Acquisition and Manipulation, Ministry of Education, Anhui University, Hefei, 230039 China; 2 State Key Laboratory of Pulsed Power Laser Technology, Hefei, 230037 China; 3 Department of Physics, Anhui Medical University, Hefei, 230032 China

**Keywords:** Metamaterials, Nanowires

## Abstract

Due to their low losses, dielectric metamaterials provide an ideal resolution to construct ultra-narrowband absorbers. To improve the sensing performance, we present numerically a near-infrared ultra-narrowband absorber by putting ultra-sparse dielectric nanowire grids on metal substrate in this paper. The simulation results show that the absorber has an absorption rate larger than 0.99 with full width at half-maximum (FWHM) of 0.38 nm. The simulation field distribution also indicates that the ultra-narrowband absorption is originated from the low loss in the guided-mode resonance. Thanks to the ultra-narrow absorption bandwidths and the electric field mainly distributed out of the ultra-sparse dielectric nanowire grids, our absorber has a high sensitivity *S* of 1052 nm/RIU and a large figure of merit (FOM) of 2768 which mean that this ultra-narrowband absorber can be applied as a high-performance refractive index sensor.

## Introduction

Metamaterials are built with artificially constructed materials which can be manipulated to produce exotic optical properties such as super-lenses^1^, negative refraction^2^, asymmetric transmission^3^, cloaking^4^ and absorbers^5^. Among them, metamaterials absorbers, which can realize near-perfect absorption by designing the nanostructures, are very appealing because they can be applied in photodetectors^6,7^, sensors^8^, thermo-photovoltaics (TPV)^9^, thermal emitters^10,11^, and solar cells^12,13^. So far, a variety of metamaterials absorbers have been proposed with different bandwidths to meet the different application demands. To achieve high performance, lots of efforts have been devoted to broadening or narrowing the absorption bandwidths^14–18^. For example, the broadband absorbers are usually designed to be applied in photodetectors and solar cells^19,20^. On the other hand, the absorbers with narrower absorption bandwidth have better performance in the applications of thermal emitters and sensors^8,21^. Up to date, some ultra-narrowband absorbers have been proposed by manipulating electromagnetic resonance in metallic microstructures^22–26^.

In recent years, it has been found that dielectric metamaterials (DMs) composed of dielectric microstructures can also be used to manipulate electromagnetic resonance^27–32^. Due to the unique advantage of the low loss, dielectric metamaterials provide an ideal resolution for narrowing the absorption bandwidth^33–35^. In addition, the ultra-narrowband absorbers were reported by using dielectric microstructures recently^36,37^. However, how to improve the sensing performance of a dielectric metamaterial absorber is still under exploration. On the other hand, it is well known that the sensing performance of an absorber can be evaluated by sensitivity $\left(S=\frac{d\lambda }{\varDelta n}\right)$ and FOM (FOM = *S*/FWHM) where $d\lambda$ is the resonance wavelength shift with the refractive index change $\varDelta n$. Thus, according to the definition, an absorber with high sensing performance should have both ultra-narrow absorption bandwidth and high sensitivity.

In this paper, we report an ultra-narrowband absorber under TE-polarization (electric field parallel to the nanowire grids) incidence in the near-infrared regime by using ultra-sparse dielectric nanowire grids. In the absorber configuration, silicon and gold are selected as the materials of nanowire grids and substrate, respectively. This absorber has an absorption rate exceeding 0.99 and the absorption bandwidth less than 0.40 nm. Furthermore, our absorber has a high sensitivity S of 1052 nm/RIU and a large FOM of 2768. The simulation also shows that, the guided-mode resonance in the dielectric structures leads to an ultra-narrow absorption bandwidth, while the ultra-sparse grids enable the electric field to mostly locate out of grids so that the strong interaction between the electromagnetic field and the analyte occurs to eventually enhance the sensitivity. This ultra-narrowband absorber can be applied as a high-performance refractive index sensor.

## Modeling Method

**Figure 1:**
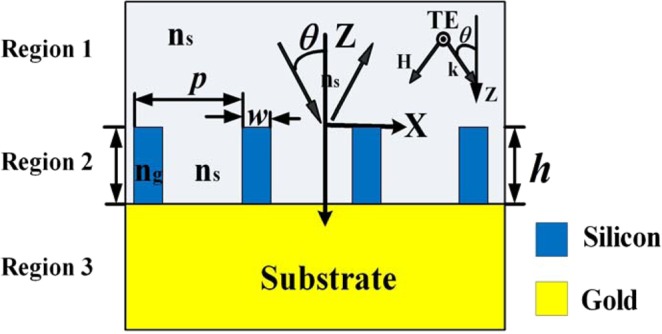
Geometry of the dielectric metamaterials.

The rigorous coupled-wave analysis (RCWA) is employed to investigate the periodic structures which are bounded with region 1 $(z\, < 0)$ and 3 $(z\, > h)$ as depicted in Fig. 1^38^. The periodic structure in region 2 $(h\ge z\,\ge 0)$ can be described by the period *p*, silicon width *w*, height *h*, and filled factor $f=w/p$. The modulated permittivity in region 2 can be decomposed as a Fourier series given by

1 $$\varepsilon (x)=\sum _{h}{\varepsilon }_{h}\exp \left(j\frac{2\pi h}{p}\right),$$

where ${\varepsilon }_{h}$ is the *h*th Fourier-series coefficient in region 2. In addition, the electromagnetic fields is expanded as a Floquet-Bloch series in the simulation process. By matching the tangential electric- and magnetic-field components at the boundary (*z* = 0 and *z = h*), we can obtain the transmitted (*T*) and reflected (*R*) light. Based on the principle of energy conversation, the absorption rate *A* can be attained with *A = 1-R-T*. In the simulation process, the transmission *T* is treated as zero because the gold substrate is thick enough to completely reflect or consume the incidence light. Therefore the absorption rate *A* can be attained with *A = 1-R*. We note that the accuracy of the calculated results depends on the number of Fourier components. If the number of Fourier components is large enough, the simulation results are stable. For TE-polarization light, to ensure the convergence and save the computation resource, a total of 101 Fourier components are employed in the simulation process^38^.

## Results and Discussion

The permittivity of silicon in the proposed structure is chosen from ref. ^39^, and the permittivity of gold in the infrared regime can be described with a Drude model:

2 $$\varepsilon =1-\frac{{\omega }_{p}^{2}}{\omega (\omega +i{\omega }_{c})},$$

where ${\omega }_{p}=1.32\times {10}^{16}rad/s$, ${\omega }_{c}=1.2\times {10}^{14}rad/s$, and $\omega$ is the angular frequency of the incidence light. In addition, ${n}_{s}$ represents the refractive index of region 1 and the grating groove, and ${n}_{g}$ is the refractive index of the nanowire grids.

**Figure 2:**
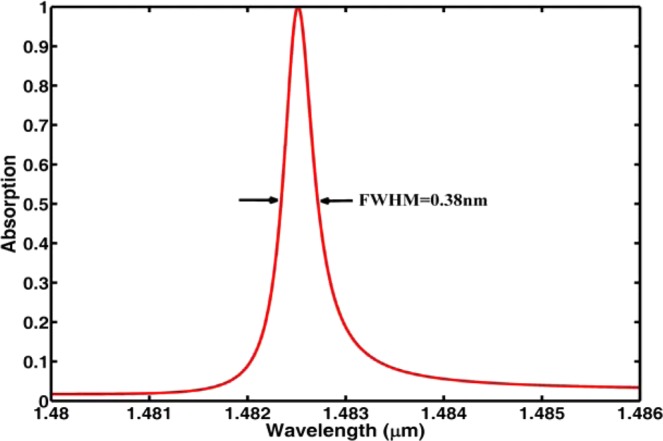
Absorption spectrum of the dielectric metamaterial.

Figure 2 shows the absorption spectrum of the proposed dielectric metamaterials for TE polarization, and the optimized parameters used in the simulation are $p=1.300\,\mu m$, ${\rm{f}}=0.02$, $w=0.026\,\mu m$, $\theta ={0}^{o}$, ${n}_{s}=1$, and $h=1.656\,\mu m$. From Fig. 2, it can be seen that there is an absorption peak at the wavelength of $\lambda =1.48251\,\mu m$ with absorption rate larger than 0.99. Figure 2 also shows that the FWHM is 0.38 nm which is narrower than those reported in^23–26,33–36^. Thus, we can get a TE-polarization absorber with ultra-narrow bandwidth in the near-infrared regime by using dielectric metamaterial. It has to be mentioned that our ultra-narrowband absorber includes ultra-sparse nanowire grids with aspect ratio of $h/w=64$ which may be a challenging task in fabrication. However, the proposed periodic structure can be realized by using Au metal assisted chemical etching^40^.

**Figure 3:**
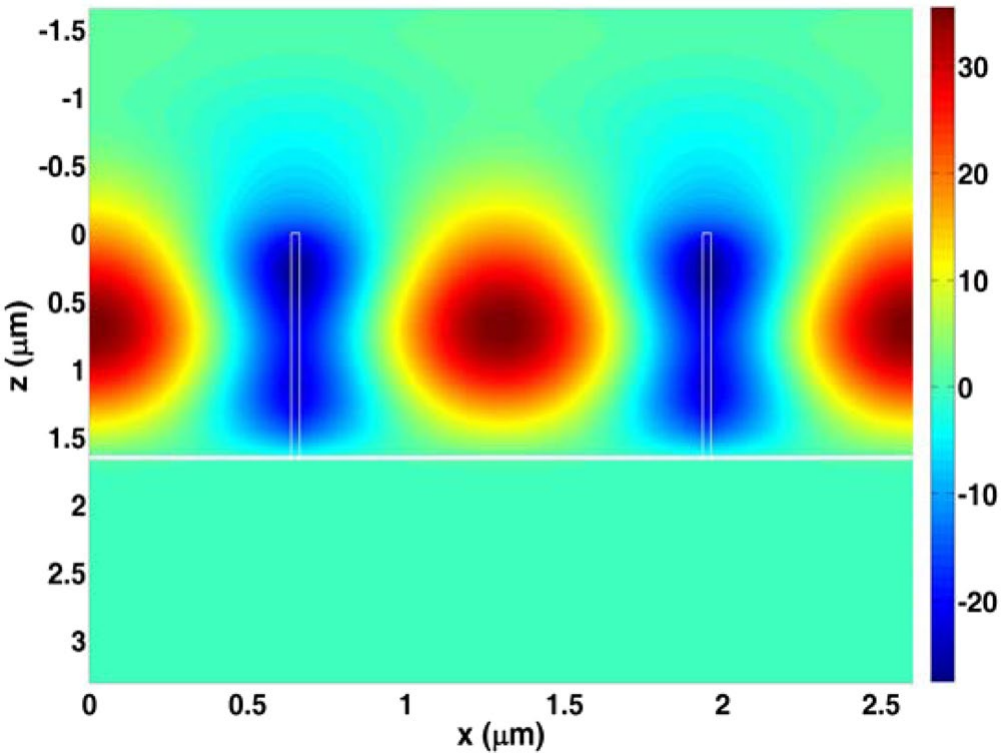
Electric field distribution at the resonance absorption wavelength.

In order to explain the ultra-narrowband absorption mechanism, the electric field distributions of two periods at the resonance absorption wavelength have been calculated in Fig. 3. In the next simulation, all parameters are the same with those used in Fig. 2 if it is not specified. As shown in Fig. 3, the obvious standing wave profile in X direction indicates that the guided-mode resonance occurs in the grating layers. To further verify the guided-mode resonance effect, we try to derive the resonance peak by utilizing the guided-mode resonance theory. According the guided-mode resonance effect, for the first-order grating diffraction, the resonance wavelength at normal incidence can be written as follows:

3 $${\lambda }_{resonance}=p{n}_{eff}.$$

where ${n}_{eff}$ is the effective refractive index of the grating region^41,42^. Furthermore, by using the effective medium theory, the effective refractive index for TE polarization can be calculated by^43^:

4 $${n}_{eff}=[{n}_{s}^{2}(1-f)+{n}_{g}^{2}f].$$

From Eqs. (3) and (4), if the parameters are set as ${n}_{g}=3.49$, ${n}_{s}=1.0$, and ${\rm{f}}=0.02$, we can get ${\lambda }_{resonance}=1.438\,\mu {\rm{m}}$ which is close to the absorption peak shown in Fig. 2, and such results also indicate that the guided-mode resonance effect occurs in our ultra-narrowband absorber.

From Fig. 3, we can also see that the electric field is localized within one period, so that one grating period can be treated as a Fabry-Perot cavity in which the cavity length is the period *p* and the nanowire grids act as cavity mirrors. According to the cavity resonance theory, the spectral width of the resonance peak can be given by:

5 $$\varDelta \lambda \propto \frac{1}{{n}_{s}p}\frac{\arctan \left(\frac{1-{\bar{R}}^{2}}{2\bar{R}}\right)}{\pi }.$$

where $\bar{R}$ is the cavity mirror reflectivity^44,45^. In this absorber design, we use the dielectric nanowire grids as the cavity mirrors, so that the higher reflectivity will be obtained. Thus, based on Eq. (5), we can explain that the ultra-narrowband absorption in the absorber is originated from the high reflectivity produced by the dielectric nanowire grids.

**Figure 4:**
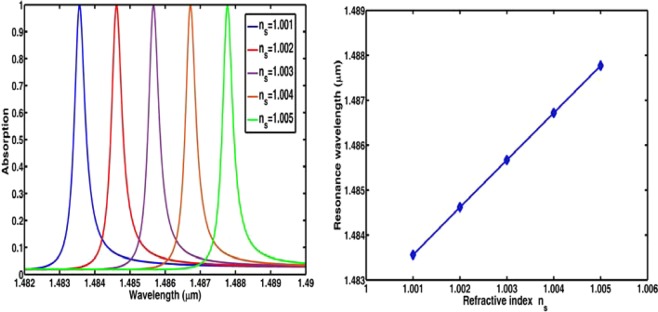
(**a**) Absorption spectra with different surrounding medium; (**b**) absorption peaks as a function of refractive index of the surrounding medium.

From Fig. 3, it can be clearly seen that the electric field is mainly distributed in the grating grooves which may provide an ideal sensing platform to enhance the interaction between the molecules or atoms injected in the grooves and the localized electric field^8,45–49^. The sensing performance of this absorber is calculated and plotted in Fig. 4. Figure 4(a) shows the absorption spectra with different ${n}_{s}$. From Fig. 4(a), we can see that, the resonance wavelength will have a red-shift when ${n}_{s}$ increases, but the FWHMs remain unchanged under the different surrounding medium. Figure 4(b) shows the absorption peaks extracted from Fig. 4(a) as a function of the refractive index of ${n}_{s}$. It is clearly seen in Fig. 4(b) that there is a good linearity between the absorption peak and the refractive index of the surrounding medium. From the data in Fig. 4, *S* and FOM are respectively 1052 nm/RIU and 2768 which are much larger than those of the dielectric absorbers reported in^34,36^.

**Figure 5:**
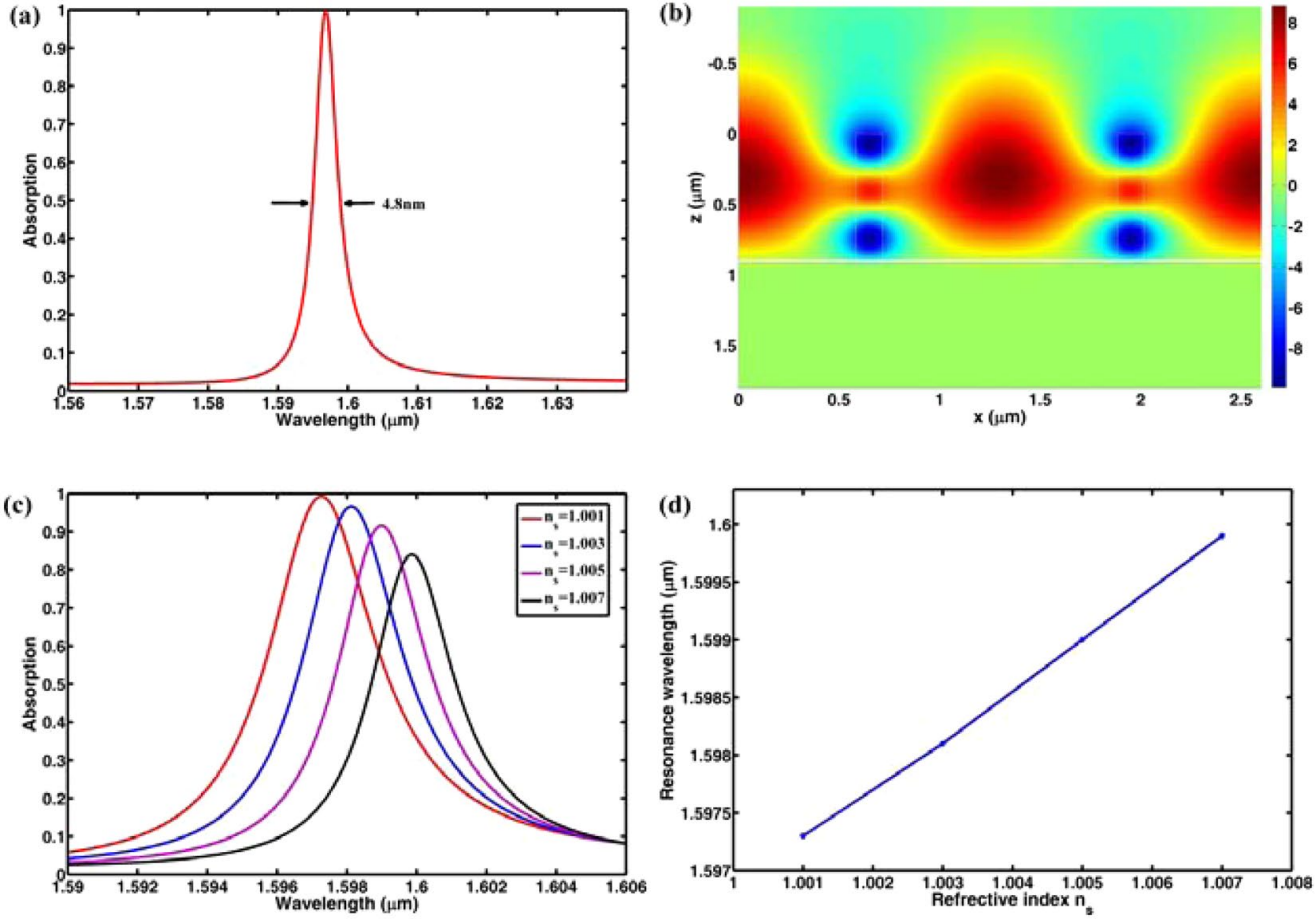
(**a**) Absorption spectrum of the dielectric metamaterial with larger filled factor; (**b**) electric field distribution at the resonance wavelength; (**c**) absorption spectra with different surrounding medium; (**d**) absorption peaks as a function of refractive index of the surrounding medium.

In order to further demonstrate the influence of electric field distribution on the sensing performance, we calculate the absorption and sensing performance of a similar structure with larger filled factor and corresponding optimized grating height. Figure 5(a) shows the absorption spectrum with ${\rm{f}}=0.10$, $w=0.13\,\mu m$, and $h=0.9\,\mu m$. From Fig. 5(a), we can see that there is an absorption peak at the wavelength of $1.5968\,\mu m$ with bandwidth of 4.8 nm. Figure 5(b) is the electric field distribution at the absorption peak. Compared with the electric field distribution in Fig. 3, more electric field is located in the grating ridges in Fig. 5(b), and such electric field distribution in Fig. 5(b) means that the cavity mirrors made up of grating ridges will have large loss. This thus causes a reduction of $\bar{R}$ and subsequently a broadening of the absorption peak. Figure 5(c) shows the absorption spectra with different ${n}_{s}$. Figure 5(d) shows the absorption peaks extracted from Fig. 5(c) as a function of ${n}_{s}$. From Fig. 5(d), we can get the sensitivity of 433 which is much smaller than that of the structure with ${\rm{f}}=0.02$. Thus, the results in Fig. 5 also indicate that the electric field distributed in the grating grooves plays an active role in the sensing performance.

**Figure 6:**
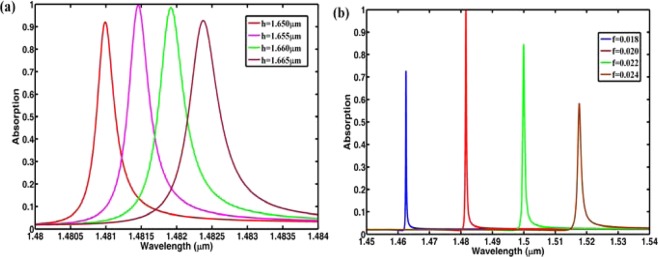
(**a**) Absorption spectra with different grid height; (**b**) absorption spectra with different filled factors.

Next, we investigate how the geometrical parameters affect the absorption characteristics. Figure 6(a,b) present the absorption spectra with the different grid heights and filled factors, respectively. From Fig. 6, we can see that the absorption peaks will shift to the longer wavelength with the increase of grid heights or filled factors. In addition, the absorption rate will decrease if the grid height and filled factor deviate from the optimized parameters, but there are still obvious absorption peaks in the observed wavelength range. These simulation results indicate that one can tune the absorption peaks through changing the geometrical parameters.

**Figure 7:**
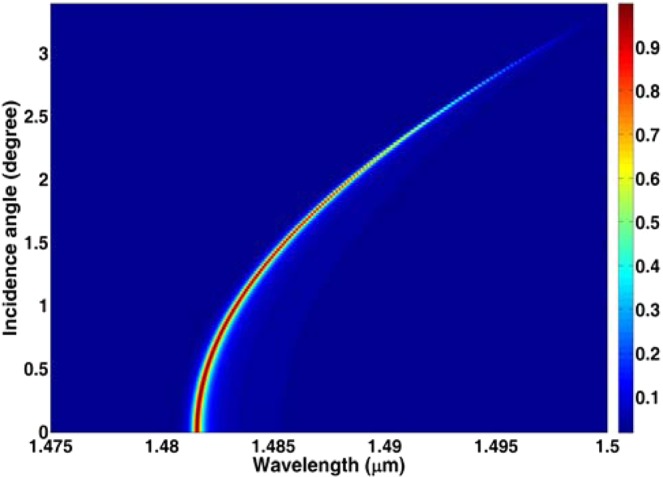
Absorption spectra with different incidence angles.

In order to investigate the influence of incidence angles on the absorption spectra, we calculate the absorption spectra with different incidence angles in Fig. 7. From Fig. 7, we can see that the resonance absorption peaks will have a red-shift with the increase of incidence angles. In addition, the absorption rate slightly decreases if the incidence angle increases, and the absorption rate is still larger than 0.80 for angles up to 2 degree. For angles larger than 2 degree, the absorption rates rapidly decrease. The simulation results indicate that one can change the working peaks by tuning the incidence angle.

From the above discussion, we can find that an ultra-narrowband absorber for sensing applications can be achieved by using ultra-sparse dielectric nanowire grids on metal substrate. According the physical mechanism, one can design a dielectric ultra-narrowband absorber with four stages. Firstly, the high-reflective metals in the near-infrared band are selected to efficiently reflect the incidence light. Secondly, the smaller filled factor is under consideration to make more field distribute out of the grids. Thirdly, according to the designing target wavelength, the parameters (*p*, ${n}_{g}$, and *f*) can be determined based on Eqs. (3) and (4). Finally, all parameters can be optimized by using RCWA.

The absorption properties of the proposed structure can be explored by the reflectance spectra. In the experiment setup, a tungsten lamp can be used as the broadband light source. The resonance absorption peaks can be achieved with a spectrometer. In addition, the wavelength shift can be tracked in real time by using a spectrometer. Thus, the measurement experiment can be realized with the current technology.

## Conclusion

We present a near-infrared ultra-narrowband absorber under TE-polarization incidence by putting ultra-sparse dielectric nanowire grids on a metal substrate in this paper. This absorber has a absorption rate larger than 0.99 with absorption bandwidth of 0.38 nm. Thanks to the ultra-narrow absorption bandwidths and the electric field mainly distributed in the grating grooves, our absorber has a high sensitivity *S* of 1052 nm/RIU and a large FOM of 2768. This ultra-narrowband absorber can be applied as a high-performance refractive index sensor.
